# Foreign

**Published:** 1840-11-28

**Authors:** 


### FOREIGN.

*De Irritide, Commentatio ab Illustrissima Societate Medico Practica quæ Lutetiae Parisiorum floret, in altero certamine die xxvii. M. Septembris, anni* 1836. *Praemio Aureo publice ornata.* Scripsit FRIDERICUS AUGUSTUS AB AMMON, Med. D., &c. Accedunt in Tabb. Aen. ii. Figg. Pictæ xviii. Lipsiæ, 1838, 4to, pp. 48. *Prize Essay on Iritis.* By FREDERIC AUGUSTUS VON AMMON of Dresden, &c. Leipsic, 1838.

(Concluded.)

In chronic parenchymatous iritis, not uncommonly little bodies or tubercles are seen in the pupillary circle of the iris and its margin, yellow or brown in colour, and various in shape and size. These may proceed from, or be connected with, either the anterior serous surface, or the parenchyma, or the uvea. In the first case they float to and fro in the aqueous humour, or they may be firmly attached to the anterior surface of the iris. When they are connected with the uvea, they generally cause synizesis of the pupil, or complete posterior synechia.

The reddish or pink-coloured circle or zone of vessels to which attention was first directed in this disease as completely pathognomonic, by John Cunningham Saunders, is invariably observed, though in different degrees. The present author regards it as not always the same in origin and nature. According to him it may be seated either in the ring of the conjunctiva, or to the extreme margin of the sclerotic coat, or in the ciliary circle, or in the venous circle of the iris.

1. The internal redness of the eye which usually accompanies iritis proceeds from the sclerotic coat, and surrounds the cornea as it were with a red zone. This is chiefly observed at the beginning of the disease; for the anterior part of the sclerotic coat, which is in contact with the cornea, assumes a bright red colour, by reason of the numerous minute vessels, which are seen through the conjunctiva, which has hitherto undergone no change. This redness in the inflamed sclerotic presents this peculiarity, that the vessels conspicuous at the termination of the sclerotic go to the cornea in a straight line, and there forming ramifications make a reddish circle round the cornea. As the inflammation of the iris increases, the redness and enlargement of the vessels also increase; the vessels of the conjunctiva, which differ from those of the sclerotic in their cochineal red colour, greater compass, and more superficial position, are dilated in the anterior part of the eye, and its most minute and most crowded ramifications being entwined with the bright red of the sclerotic coat, increase the red circle round the cornea.

2. The cornea is sometimes surrounded by a red zone, the vessels of which disapper suddenly at its margin, and assume more brilliant colours towards the internal part of the eyeball. In acute iritis the tint of this zone is deep red, and, according to the degree of the dilatation of the vessels, the rest of the surface of the eyeball is of a reddish colour. This vascular circle depends both on the annulus or ring of the conjunctiva, and also on the ciliary circle, which is very often attacked with inflammation either before or along with the iris.

3. The origin of the bluish circle which sometimes surrounds the cornea in iritis is different. The part of the sclerotic nearest to the cornea shows the circle filled with venous blood.

It is scarcely requisite to dwell on the subjective or rational signs of iritic inflammation. The most urgent are, intolerance of light (*photophobia*,) pain of the eye, and various degrees of weakened vision.

Iritis almost always continues for several weeks, sometimes for months; as it rarely terminates in resolution, if it have proceeded to its greatest and most intense degree; and its terminations are always dangerous to the integrity of the organ. Besides synizesis and different forms of synechia, and other changes already mentioned, iritis is liable to terminate in atrophy of the eyeball, or rarefaction of the iris, and other changes.

Iritis may be followed by more or less atrophy. If the cornea is affected with onyx or hypopyon, not uncommonly the cornea is ruptured, the humours escape, and complete atrophy of the eyeball ensues. In atrophy following iritis, after the inflammation is over, the eyeball appears smaller than natural. In such an eye the cornea is not round, but almost oblong; the iris is dull; the pupil not altogether round, but here and there angular, and tending upwards. The union of the cornea with the sclerotic is not natural; for at the spot at which the former passes into the latter, it forms a white circle more or less similar to the *arcus senilis.* A circle of this kind the author ascribes to inflammation attacking the ciliary body with the iris, and causing in the former condensation or atrophy. The anterior figure and aspect of the eyeball is changed, and the natural size of the eyeball is diminished.

Atrophy, or, as the author terms it, rarefaction of the iris, (*Iridaraesis*,) but which should probably be denominated attenuation of that membrane, is an affection of rare occurrence. In this disease, which the author has seen in blue or azure-coloured eyes only, the proper tissue of the iris becomes thinner than in the natural state, though whether from diminished secretion or increased absorption he does not know. The motion is either diminished or destroyed; its colour is pale, and as if dead; and the pupil is small, and its margins are irregular. The black pigment is more or less deficient, or it is so pale as to give the iris an ash-gray colour; and in one case the author observed a want in the substance of the membrane near its pupillary margin. This rare affection appears to be chiefly the result of chronic irritation, and is mostly observed among persons advanced in life; yet several times the author observed it in strumous children, whose eyeballs had been wounded. With it is sometimes associated *Iridodenosis*, and sometimes cataract.

Another consequence of chronic iritis is *Iridodenosis*, or the wavering iris—that wavering motion in which, according to the motions of the eyeball, the iris is agitated to and fro towards the cornea, or towards the lens, without change in the pupil. This wavering motion sometimes presents the appearance of tremor, when it is difficult to tell whether the tremulous motion affects the whole parenchyma of the iris, or individual parts of the membrane. With this wavering of the iris, which must not be confounded with *hippus*, (*Nystagmus Bulbi*,) or the wavering motion of the whole eyeball, greater or less opacity of the lens, or even a floating cataract may be associated.

Iritis is also liable to terminate in cataract, especially when the inflammation affects the anterior part of the capsule, forming *Irido-periphakitis*. As this rarely ends in resolution, the usual result is opacity of the capsule. Dr. Von Ammon, however, says that the opacity of the anterior wall of the capsule is not the effect of the inflammation of the capsule, but that it arises from contact of the coagulable matter secreted upon the uvea or the margin of the pupil. This form of cataract is not incurable; and the physician who understands well the variable nature of this disease will cure it most readily. That form of cataract, however, is incurable which arises from inflammation of the iris, complicated with that of the choroid coat; and which is usually glaucomatous.

Among other terminations of iritis the author ranks inflammation of the choroid coat and retina, in consequence of inflammation extending from the iris to these tissues, constituting bad forms of amaurosis, and also hydropthalmus and staphyloma of the sclerotic coat.

Lastly, the author adds, that when the iris has been once inflamed or injured by inflammation, it is very liable to be so again, and the subsequent attacks are always more injurious and more difficult to control or subdue than primary ones. It is then that not only is the inflammation liable to extend from the iris to the other tissues of the eyeball, but it is also liable to produce various new morbid growths, as hydatids, ossification, and fungous growths.

In speaking of the treatment required for the cure or alleviation of iritis, the author first observes, that the curative powers of nature are feeble, or rather altogether impotent. Every attack of iritis is difficult to be cured; every attack tends to the disorganization of the iris, or the connected parts of the eye; and the most energetic measures require to be promptly adopted, in order to avert, as far as may be, the bad consequences. Dr. Ammon well observes, that there is in iritis no cure except resolution; and, consequently, every thing which does not tend to promote this termination—every thing which does not contribute to abate or remove inflammation, without effusion of lymph, purulent matter, or other morbid products, has the effect of destroying the organ. The terminations of inflammation in other textures and organs of the body, in effusion of blood, lymph, or purulent matter, and which do not in all cases unfit the organ for its functions, are here most prejudicial, for, on the one hand, they limit, impair, or destroy the motions of the iris; on the other, they obscure vision, by destroying the translucency of the textures; and, thirdly, by adhesions they render the pupil so small that it does not admit sufficient light, or they may close it altogether, forming *Atresia.*

The author forms two indications of cure; one to diminish and remove inflammation; the other to remove the sequelæ or consequences of the disease.

To accomplish the first indication, the most important means are, copious and repeated general blood-letting, with other divisions of the antiphlogistic regimen. Dr. V. Ammon recommends general blood-letting on the first day of the disease until the pains abate. The misfortune is, that, in employing this remedy, it is often impossible to tell the first day of the disease; and in almost all cases patients apply for assistance, not on the first day, but after the disease has been proceeding for days, or even weeks, and the pain in the eyeball and intolerance of light are no longer endurable. In the meantime, however, irreparable mischief is often wrought within the eye. It is, neverless, always prudent to employ blood-letting, even when morbid products are in the act of forming, because, by subduing the inflammation, it not only puts a stop to the secretion of these morbid products, but it gives some chance, however slight, of effecting their obsorption. Dr. V. Ammon thinks that in slight cases of iritis the application of leeches over the temples or the mastoid processes may be sufficient, with the caution to allow the blood to flow for some hours after the leeches have dropped off. There is no doubt that this mode of depletion is useful as an auxiliary; but they must be slight cases, indeed, in which such depletion has much effect, or produces so much impression on the disease as to act as a permanent means of cure.

Besides the co-operating antiphlogistic power of eccoprotic laxatives and cathartic medicines in general, Dr. V. Ammon properly recommends the rigid observance of low, unstimulating diet, and the active use of derivative or revellent agents. Of these the most efficacious is unquestionably blistering the temporal or posterior auricular regions repeatedly, or the use of the caustic or the cautery in the cervical region, or between the shoulders. Of the seton he disapproves rather strongly, as calculated to aggravate febrile symptoms.

In baths, warm or cold, and in all external applications, he places little confidence, and recommends little confidence to be placed.

A long list of internal remedies has been mentioned as useful in curing iritis; and hemlock, leopard's bane, senega-root, meadow saffron, sarsaparilla, tartarized antimony, golden sulphuret of antimony, muriate of baryta, calomel, corrosive sublimate, red precipitate, hydriodate of potass, and hydriodate of soda, carbonate of soda, carbonate of potass, volatile oil of turpentine, and cod-liver oil, are arranged in this list.

None of these agents, Dr. Von Ammon thinks, have much real influence over the inflammatory process in which the disease consists; but he allows that they may, by exciting absorption, act in removing some of the *sequelæ* of the disease, and may also be beneficial in removing or rectifying the discratic condition which disposes to its formation. He therefore cautions the practitioner against placing any reliance on them, and impresses on him the necessity of forming a correct diagnosis, and then he will be most likely to treat the disease successfully.

On the alleged specific powers of mercury or any of its preparations he says nothing.

The traumatic form of iritis, or that which is the consequence of wounds or injuries of the eye, the iris, or some connected tissue, which is considered in the third chapter, differs from the idiopathic form of the disease in no respect, unless in its appearing more speedily after the application of the exciting cause, and in its proceeding more rapidly to the formation of morbid products and disorganization of the eye. It hence requires the more prompt and energetic use of remedial measures.

In chapter fourth the author enters on a full and detailed account of the serous form of iritis, or that which affects the anterior surface of the membrane. It is chiefly distinguished by its connection with, or its liability to pass into inflammation of the cornea, (*keratitis*,) and the consequent early appearance of the cochineal or pink-coloured ring or zone round the union of the sclerotic and the cornea.

This form of iritis Dr. V. Ammon represents very rarely to take place in the state of health; that is, it is most usually observed in cachectic persons, and as a consequence of wounds of the eye in those labouring under the strumous, the rheumatic, or the psoric distemperature. It is hence observed after operations for cataract, especially after keratonyxis or the anterior operation; and the author has observed it very commonly among scrofulous infants, following either measles, scarlet fever, or small-pox, true or modified. Among adults it is observed after chilling; but all such patients labour under the disposition to rheumatism, and to itch, (*psora*,) or the pining decay of inveterate syphilis. We have already mentioned what we conceive to be the most natural explanation of this; and we may here add, that the disposition to serous iritis is often induced by the operation of mercury on the economy.

Dr. Von Ammon distinguishes this variety of iritis into sero-strumous iritis, sero-rheumatic iritis, and sero-cachectic iritis.

The fifth chapter is devoted to the subject of the parenchymatous iritic inflammation. Under this head the author distinguishes two general forms; the simple parenchymatous iritis, consisting of the gouty and the syphilitic iritis;—and the complicated parenchymatous iritis, consisting of syphilitico-mercurial iritis, syphilitico-arthritic iritis, syphilitico-scorbutic iritis, scrophuloso-syphilitic iritis, scrophuloso-psoric iritis, and the iritis scrophuloso-plicosa. These varieties are described chiefly with a view to variations in treatment. Our countrymen will perhaps think that Dr. Von Ammon has been too minute and refined in his distinctions. But in answer to this, we must recommend them to study attentively his distinctions, and consider carefully what he says in their behalf.

Inflammation or the uvea is considered in the sixth and last chapter.

The most remarkable circumstance in this variety of iritis is the swelling of the whole iris, so as to protrude the membrane forwards into the anterior chamber; and its tendency to produce opacity of the capsule.

This form of iritic inflammation the author ascribes to the operation of the gouty, the strumous, or the syphilitic distemperature; to the state of the system induced by the puerperal condition, to abdominal plethora, and hepato-portal congestion, when there are generally hæmorrhoidal symptoms, external or internal; and, in short, to all those causes which disorder digestion and vitiate the fluids.

The treatment recommended is very similar to that advised for serous or parenchymatous iritis. But the author recommends strongly pure air, regulated diet, local blood-letting, by leeches or the cupping-glass, the use of the revellents, diaphoretics, the free use of opiates when pain is very urgent, and the employment of the thermal baths of Carlsbad, Marienbad, and Egerbad.

We must not dwell longer on this essay. It is sufficient to say, that, as a monograph on iritis, it is a very masterly performance, and shows that the author has studied the disease most attentively, is intimately acquainted with its varieties, and is an opthalmologist of great skill and judgment.—*Edinburgh Med. and Surg. Journal.* October, 1840.

---

*Cases of Spontaneous or Idiopathic Emphysema.* By James Mouat, M. D.—The evolution or secretion of air from the blood into the cellular tissue is a very rare phenomenon. Indeed, so few cases are there detailed by medical authors, that I am induced to record a recent instance of spontaneous or intrinsic emphysema occurring in India.

Private William Preston, H. M. 13th Light Dragoons, aged 32½, in India seven years, a tailor by trade, dark hair, spare make, swarthy, but healthy, was in hospital with symptoms of *dysenteria ac.* 4th July and 14th August; and *hepatitis acuta*, 17th August, 1837; since which period he has been at his duty, and working in the tailor's shop till the evening of the 9th of June, 1839, when he was admitted with pain and oppression in the chest, and slight cough, attended by severe headach; heat of skin, succeeded at times by cold chills; much thirst; pulse quick and hard; tongue foul and loaded; bowels regular. He had been ill two days previous to admission, and can assign no cause for his ailment.

He was immediately bled to thirty ounces, and suitably treated with purgatives, the *Solut. Antimon. Tartarizat.* &c. with scarcely any relief. There being considerable fever the following morning, the blood-letting was repeated to twenty ounces. In the evening he still complained of much pain in the chest, with some dyspnœa, and quick respiration; pulse one hundred; much thirst. The bowels had been freely evacuated. Thirty leeches were now applied to the seat of pain, and the saline remedies persevered in with so much relief that he had no cough or accession of fever. The pulse became soft and regular at eighty; tongue foul and loaded. On the 12th, half-past six A. M., reported better; no fever; slight cough, no expectoration; tongue foul; no stool; but seems gettting better. Was seen by Mr. Parrock at the visit, who especially noticed this patient. 10 A. M. For the first time, has complained of severe shooting pain all over the right thigh, particularly towards the groin, where there is slight swelling, with much heat of the parts. The vessels of the thigh appear natural and soft; states he first perceived this pain, rather suddenly, about half an hour ago, before which he was quite easy; pulse quick; skin warm; tongue clean; bowels open twice; appears rather anxious. Twelve leeches were applied to the parts affected, followed by fomentations. 12 Noon. Leeches have just come off, and states he feels somewhat relieved; not so restless; vessels of thigh natural, but hot; distension in groin the same; pulse quick; skin warm; appears anxious. The fomentations were continued.

Half-past 2 P. M. Has become suddenly much worse; looks anxious, and nearly of a leaden hue; quick convulsive respiration, deep and almost stertorous; great oppression at præcordia and about the heart; also feeling of pain all over him, and particularly of right thigh, which is now much swollen and distended, from the hip to the knee; hard, tense, and somewhat discoloured above, but softer and elastic, and crepitating towards the knee; starts in bed at times; pulse quick and irregular, one hundred and twenty; chest sounds well, and the stethoscope reveals a curious gurgling kind of *bruit de soufflet*; but the respiratory murmur is natural, and there is no abnormal sound about the limb; pulse at groin vibrating or bounding, and no pulse in the ham. One long incision was made outside the thigh, and another near the knee, about one inch in length, which gave vent to a reddish serosity with bubbles of air.

℞. *Æther. Sulph.* ʒss. *Tinct. Opii.* m. xx. *Mist. Camphor.* ℥i. *M. stat. sum. et repet. om. sem. hor.—Frictio.*

3. P. M. Little or no hæmorrhage from incisions, but a great deal of air escapes in bubbles, with some sanious fluid. This relieved him a little, but the restlessness continues, with short quick respiration, and he is becoming weaker and weaker, and more oppressed, and the limb more swollen, painful, and distended, and on pressure much air escapes from the incisions.—*Cont. remedia.*

Half-past 3 P. M. Pulse weaker, and a fainting sensation at times; some relief from the draught; limb the same; seems worse; more anxiety; and great distress of countenance.—*Cont. remed.*

Quarter to 4 P. M. Sinking fast; cold clammy sweats; respiration more laborious; pulse nearly gone; still air in bubbles come away from the incisions.—*Cont. remed.*

Died five minutes to 4 P. M.

*Sectio Cadaveris* one hour and a quarter after death, in the presence of Messrs. Smith, Kennedy, Coleridge, and Parrock.

*Head.*—On removing the *dura* and *pia mater* about six ounces of serous fluid escaped; a few bubbles of air were observed on the surface of the *pia mater*. The brain was softer than natural; about three drachms of pale serous fluid were contained in each lateral ventricle.

*Thorax.*—Left lung collapsed; the right lung had old adhesions to *pleura costalis*, and both lungs appeared healthy. About two ounces of serous fluid were contained in pericardium, and some air bubbles, like vesicles, on its surface. The heart was pale, flabby, and collapsed, with several small bubbles of air on its upper surface, the size of millet-seed. The heart was carefully removed, and the vessels compressed, in order to exclude the external air, and immersed in water, when some air rose to the surface from the right side. This viscus was found nearly empty, and contained sufficient blood only to tinge the fluid of a light red colour.

*Abdomen.*—Liver rather larger than natural, more brittle in texture, and of a pale dram-drinking appearance. Great hypertrophy of the spleen, which had increased to nearly twice its natural size, and was of a dark purple colour. A few ulcerated spots were observed in the large intestines. The mesenteric glands were enlarged. The other viscera were healthy.

The right thigh was much enlarged, hard, distended, shining, and somewhat discoloured, when punctured giving out much gas. On turning back the skin and fasciæ, the muscles were seen of a dark purple colour, commencing about the origin, and extending half way down the thigh, and when incisions were made into them, a quantity of air and fluid blood escaped. The blood and air seemed to be pretty equally diffused throughout the whole tissues of the muscles, appearing like clotted blood, giving a crepitating feel like lung, but appeared to be more abundant in the extensor and abductor muscles. The Sartorius muscle free from disease, but much swollen. The blood-vessels were healthy. The femoral artery and vein, with their large branches, were carefully dissected. The cellular tissue of the large blood-vessels was infiltrated with dark blood, and bubbles of air accompanying the vessels under Poupart's ligament to the fore part of the thigh. The femoral vein contained some air; all the other vessels containing merely fluid blood. Much air issued, with frothy sanious fluid, when cut into and exposed. It appeared to have transuded and given out the air or gas.

The remarkable features of this case during life were its sudden and unaccountable appearance; the rapid sinking and great distress in the course of its brief career; the sudden swelling, and crepitating feel of the limb; the issue of bubbles of air, with a watery serosity, when the distended parts were incised; the leaden hue and great anxiety of countenance, &c. The important circumstances disclosed by dissection were bubbles of air on the surface of the *pia mater;* the softening of the brain; air bubbles like vesicles on the pericardium; the heart pale, flabby, and collapsed, with air globules on its surface, and air contained in the right side of the heart; the enlargement of the diseased limb giving out gas when punctured; the dark purple colour of the muscles also containing air and fluid, and its diffusion through the connecting tissues of the muscles, giving them the appearance of hepatized lung, as well as crepitating; the cellular tissue of the large vessels infiltrated with dark fluid blood, and vesicles of air; the femoral vein containing gas, but the other veins, fluid blood, and their tissues, a frothy sanious fluid, which appeared to have transuded, and given out air or gas. The femoral vein and diseased muscles were removed, washed, and then put into water. When examined twelve hours after, they were increased in bulk, and covered with bubbles of air, and still gave the crepitating feel of lung. The small quantity of air preserved, a part had a light put to it without any very evident apparent result; the other portion with lime-water indicated carbonic acid gas.

Did the extrication of gas arise from secretion or spontaneous evolution? or from partial decomposition and alteration of the circulating fluid? Its local origin, and total unconnection with respiration, and altered state of the blood, as if deprived of its vitality, would countenance the latter supposition, arising, perhaps, from this morbid state of the vascular action, connected with depressed vital powers, giving rise to its apparent secretion, and, had the patient lived long enough, might have become a case of humid gangrene. The blood had lost its property of coagulating, giving rise probably to its tendency of extricating gas, as it seemed to transude with bubbles of air, which continued even after dissolution.

The immediate cause of death may have arisen from air getting into the circulation, as the heart was pale, flaccid, and flabby, and had air in its right cavities.—*Ed. Med. and. Surg. Journ.*

*Bangalore, East Indies, June,* 1839.

---

*Practical Observations on the Diseases of Peru, described as they occur on the Coast and in the Sierra.* By ARCHIBALD SMITH, M. D.

I.—DISEASES OF THE COAST CONTINUED.

*Colic.*—This is a common complaint among young and old, as a consequence of irregularities in diet, in a country where great watchfulness in diet is every day essential for the preservation of the general health.

Those who are more remarkably liable to be attacked with colic during the cloudy and wet season in Lima, are the Serranos, or inhabitants of the hills, who come to the coast at this time to trade; while on the hills it is the dry season, and the roads are hard and passable for mules and cargoes, and the weather such as to offer no risk of damage to goods, from swollen rivers, rain, or snow storms, &c. The colics of such persons, who usually put up in the "tambos," or inns frequented by muleteers and traders, are often brought on by eating olives, pears, and similar articles, to which they are not accustomed at home, and may in general be removed by a dose of castor oil, with twenty drops of laudanum, or by an opiate pill followed by one or two purgative clysters. But during the hot months, when, from the effects of increased solar heat, the excitability of the system, and especially of the gastric organs, is proportionably augmented, we have frequent occasion to witness among the regular residents in town, most severe cases of colic, very likely induced by eating curd with molasses, (of which the lower orders are very fond,) or an ordinary sweet-cake, prepared of coarse sugar, called chancaca, studded with "mani," or ground-nuts. But the most common exciting cause of all at this season, is the great consumption of melons, and of the fruit called pepino, vulgarly Mata-Serrano, or mountaineer-killer, which are considered very usual causes of fevers, colics, cholera, and diarrhœa. About vintage time in Ica, the Indians from the interior are known to suffer severely from such ailments, when they visit that part of the coast, from the great relish with which they eat the melon and half ripe grape. I shall now give a few particular illustrations of colic.

1. In Lima, when the men are sick, the women usually are *relatores*, or narrators of their case; but when they are themselves ailing, sometimes the men become narrators in turn. Thus, one man standing by the patient's bedside, said to me, when desiring to account for a severe colic, "My wife is very choleric. For a bilious movement she had experienced, she took iced pine-juice, and it agreed with her perfectly. On the day following she took iced milk, and it caused a most severe colic, from which she still suffers." This case required a purgative; and one of manna and rhubarb, in an infusion of senna, afforded relief.

2. Another man related of his wife:—She is very choleric; she frets without provocation, and the result is a fearful colic that would soon kill her, if she did not take care to drink warm water, and so produce vomiting; for as soon as she becomes choleric, the food on her stomach ceases to be digested, and becomes corrupt. Hence, at whatever hour, by day or night, she happens to be angry or peevish, she herself, to avoid a violent colic, instantly drinks warm water, by the aid of which she vomits, and is quite well again.

3. A middle-aged lady, of stout form, but nervous temperament, for some time subject to colic complaints, exceeded at supper in eating pease and rice. And next morning she sent for me, and related her own case thus:—"Over night I was seized as if it were with *lipyria.* I had violent pains in the belly, acid eructations and retching, but no actual vomiting. At the pit of the stomach I had a most violent palpitation, and so great a *fatiga*, that I looked for death; but at length evacuations came on, which carried off the rice as entire as I had eat it. Now I am at ease, and in course of the night I was visited by the catamenia."

4. A respectable white woman, of a nervous habit of body, experienced in the morning of a day in February, some annoyance that ruffled her temper; at four in the afternoon she dined, and by way of dessert after a simple meal, eat curd with honey or molasses—"*requeson con miel.*" Having finished dinner, she was again put out of temper, and from five to six in the evening, or about an hour after dinner, a vehement pain fixed itself at the seat of the stomach, from whence it soon extended to both sides in the line of the lower ribs, towards the loins, and to all the abdomen. The arms, to her sensations, became as hot as fire, the neck stiff and uneasy, and the head stupefied. Nausea and flatulency, followed by some vomiting of bitter matter, came on about seven o'clock at night, and immediately she drank warm water freely, by the aid of which she rejected curd, and all her dinner, so that she was thus relieved of her colic or cramp in the stomach, and, feeling exhausted, she slept for a little after.

During her conflict with the disease, her attendants anointed the abdomen with almond oil, and administered a couple of clysters of linseed water, which returned, carrying off very little feculent matter. After her short sleep, she awoke in a state of moderate reaction, and she now observed quietude of mind and body, used spare farinaceous food, and drank whey mixed with linseed water, till two days after the attack, when, feeling only a sense of oppression at stomach, she took, with great benefit, iced pine-juice to complete her cure.

5. In the ordinary and regular colic, neither attended with, nor ending in vomiting or evacuations, after the severe pains, unaccompanied with fever, felt about the umbilical region, had lasted a while, say several hours or a whole night, I have known the symptoms become rapidly inflammatory, so as to affect the general circulation, and give rise to a febrile state of the pulse. Blood-letting, followed by the warm bath, sinapisms to the belly, and efficient purgative medicines, aided by an emollient clyster, are in such cases approved means, which serve, in general, to arrest the progress of the disease; and the remaining irritation and tenderness may be removed by employing panada, diluent drinks, and the favourite *agua de pollo*, or chicken tea; not omitting the usual unctuous applications to the belly, which, after being anointed, the natives cover over with the warm omentum or caul, *redano*, of a sheep or pig.

6. But when this intense and inflammatory sort of colic is neglected in its origin, it may terminate in *iliac passion*, or what is commonly called

*Colico-Miserere.*—This formidable disease, which is sometimes occasioned by an *empacho* from eating cheese, like other affections attended with vomiting, the old native practitioners attack with iced drinks and cold enemata; and I believe that the latter especially may often be of service, where the former would be objectionable. Thus, I have considered the iced drink contra-indicated whenever I found it ordered to a patient with an habitual enlargement of the liver and spleen sympathizing with the inflamed intestine, and passing quickly from the chronic to the acute state of morbid action, and irritated more and more by every repetition of vomiting, or straining and retching.

We are, however, fortunately not without a remedy, which, if applied after the timely use of the lancet, may be considered, I think, less exceptionable in such cases than *ice* can be. This is calomel, in a full dose of from sixteen to twenty grains, alone or with opium, for an adult. The existing prejudice in Lima against the seasonable use of this remedy is still great, and I never saw it employed in the cases of iliac passion that I have known, from time to time, occur there.

Of the valuable sedative effects of this remedy, Sydenham, though perhaps unwittingly, has handed to posterity a practical lesson, in his treatment of a case of iliac passion, recorded in his *schedula monitoria de novæ febris ingressu.* When the violent irritation of the bowels did not yield to an enema of tobacco smoke,* he prescribed as a cathartic somewhat more efficient, *Pil. ex duobis*, gr. xxiv.; *mercur. dulc.* scrupul. i. made into pills, and to be taken in a spoonful of syrup of violets; at the same time the patient was enjoined to drink nothing, lest the pills should be rejected. Modern experience shows that sixteen or twenty grains of calomel, when given alone at a single dose, are well calculated to allay vomiting, or gastric and intestinal irritation, while the other ingredients in Sydenham's prescription, of which the *pil. ex duobis* consist, (viz. colocynth and scammony,) never, I think, could, without the aid of a sedative of some sort, be made to sit quietly on the stomach in cases of high excitement, pain, and vomiting, such as Sydenham so graphically describes.

*Cholera.*—This disease, which occurs on the coast of Peru at all seasons, is a very prevalent one in Lima during the warmer summer months, when the languor and debility of the muscular system is increased, and when the digestive organs and nervous system are most irritable and susceptible. In Ica, which is a vine district, from fifty to sixty leagues to the south of Lima, cholera is a well known disease, but not so formidable as the variety of the same which appears especially in Palpa, a coast-town twenty leagues south of Ica, land-locked among sandy hills, and where the rays of the sun are strongly reflected and concentrated. Here the disease is called *mal de ansias* or *vomito seco*, on account of the dry retching that accompanies the attack. The *mal de ansias* commences with a feeling of sickness at stomach, and a fruitless desire or effort to vomit. But this stage of painful effort, or, as it is called, of *dry vomit*, is usually followed by real vomiting and evacuations, sometimes with, at other times without, spasms of the limbs.

We are informed by an intelligent native from Ica, that the irritation is greatest, and spasms most apt to occur in those, whom the disease happens to attack with a full stomach or loaded bowels; whilst all the symptoms are milder in the individuals who are taken ill with their bowels in a less embarrassed condition.

Those who are thus attacked in Palpa, and still farther south at Acari, in which localities the disease is endemic, know it to be their safety to get as quickly as possible into the cooler air of the open sea-side; and such is the extraordinary irritability of the gastric organs under this complaint, that the natives are impressed with a practical belief that the person who tastes aught but cold water or iced water while it lasts, must die in consequence. But in Ica, where the air is less scorched, the disease is so far milder, that those affected with it venture to add a little cream of tartar or tamarinds to the water. The disease is said to be usually one of eight or more days' duration.

In the Memoirs of General Miller, we find it related at page 45 in volume second, that the General (then Colonel) suffered an attack of this disease when at Acari. The following extract bears on our subject. "At this juncture, Colonel Miller was disabled by an attack of *mal de ansias*, a species of cholera morbus. He was carried in a litter across the desert seven leagues, to the Port of Lomas, where the zealous Captain Nesen was waiting with the Protector. He was hoisted on board more dead than alive. The dreadful disorder came on every other day, and continued in paroxysms of fourteen or fifteen hours, for the space of ten days. Cold water was the only remedy administered. The complaint is common in that part of the coast; but although excruciatingly violent, not one out of three or four fall victims to it. The invalid was soon so much reduced that he spoke with difficulty. His friend Dr. Cordova, (now Dean of Arequipa,) lay ill of the ague. Both were cooped up in the state-cabin, if so it might be called, of the brig, and neither could move from his bed."

It is to be observed that agues infest the whole coast of Arequipa, from Acari, southward; and that the *mal de ansias* is not of itself a disease to appear in quotidian or tertian paroxysms. Colonel Miller's case, therefore, was one of a complicated nature, in which a *terciana*, more or less obscure, associated itself with the distressing retching characteristic of the *mal de ansias.*

The symptoms of cholera, as the disease usually appears in Lima and its environs, come on without previous warning. At times the attack comes on when at table, at other times in course of the night, or in the morning early, when the food taken on the preceding evening is brought up in a very undigested state. After a fatiguing ride in a hot day, an individual comes home in the evening, takes some refreshment, very probably eats melon, and an hour or two after he is seized with symptoms of cholera. I have known an attack of this malady follow the sucking of the juice from a piece of succulent sugar cane; and when a

* Tobacco smoke seems to prove cathartic or laxative by removing constriction,—for of the very remarkable power which tobacco infusion of even moderate strength (say ten or twelve grains infused for ten minutes in six or eight ounces of boiling water) possesses, of producing, when injected into the rectum, muscular relaxation and general depression, every surgeon has evidence in cases of incarcerated hernia.

person happens to have taken milk, or any article of milk diet, and after this, in course of the day, commits the indiscretion of tasting some acid fruit, the cause is so generally recognized as the source of gastric disturbance, that no one is surprised to hear of cholera as its consequence. It follows a fit of drunkenness; and it also frequently occurs that one has had a fit of anger, or some vexation, in course of the day, and as a sequence there is, over night, an attack of cholera, with well-marked signs of indigestion. In short, when there prevails such a predisposition to the disease as is common in summer weather, any such incidental cause as now mentioned I have known produce an attack of cholera. But indeed, without any specific occasional cause, either discovered or assigned, I have known the disease come on very suddenly and violently; and it appeared to me that women were more subject to it than men.

I shall offer a few examples to illustrate the nature of cholera, as it familiarly presents itself in Lima.

1. A little girl eat rice* at dinner, drank after it iced water, and was suddenly seized with symptoms of cholera. One hour after the commencement of the attack, I saw her in a state of insensibility, with a death-like coldness of the extremities, the features contracted, the eyes upcast, and the white alone visible; respiration was short and panting, and there were from time to time convulsive twitchings of the extremities. The watery evacuations had not yet commenced; but by this time she had vomited all her dinner, and remained quite exhausted, as described, to the alarm of those interested in her recovery. Under the plan of treatment pursued, which shall be mentioned by and by, the disease was very soon arrested in its course, and the child did well.

2. An elderly lady, who, two or three days before, experienced some headach, on a December morning, hot and sunny, walked some distance in town, from San Pedro to Santa Clara; and at night was seized with distressing colic pains, which were soon followed by vomiting, and the consequent appearance of brain-fritters and kidneys, "*torta de sesos y unos rinones*," which she took to dinner, and which were vomited in a sour and half-digested state. The belly was anointed with warm almond oil, with a view to relieve griping pains, with vomiting and purging of liquid matter, of colour and appearance very like dark chocolate, attended with cramps in the lower extremities. What she vomited, she reported to have been as bitter as gall, and what passed by stool felt scalding hot. She drank freely of tepid water, and tepid emollient clysters were repeatedly thrown up. Thus she passed the whole night, till towards morning, when the symptoms gave way; she fell asleep, and awoke without any further return of the complaint.

3. I have seen a nervous and delicate-complexioned young lady, who was habitually given to chew ice to brace her nerves, four hours after being seized with symptoms of cholera. I was told by her sisters in attendance, that, two hours after having finished her meagre Friday's dinner, she was taken with evacuations of the appearance of chocolate, and soon after came on vomiting, by which she rejected, in an undigested state, all that she had eaten at dinner. The evacuations and vomiting, I at my first visit observed to be still distressing; and she complained of much oppression at the præcordia, with palpitation and sense of weight and oppression, "fatiga," at the seat of the stomach. The skin was cold, the pulse small and frequent; her anxiety and general restlessness were very great, and she kept exclaiming, Muero! Muero! I am dying! I am dying! Next day she felt quite recovered from this attack.

4. In time of Lent, an elderly woman, four hours after dinner, consisting of fish, onions, and white peas, with some fruit, found herself taken ill with pain at stomach, and soon became very drowsy and nearly insensible; but when evacuations commenced, she was again quite sensible. I saw her five hours after the attack commenced, with her skin cold and dry, the tongue parched, and her stools, which had the appearance of turbid water, were repeated about every ten minutes. The retching or efforts at vomiting were severe, but fruitless; the pulse quick, and hardly perceptible; the cramps at stomach excruciating, and the patient called out Mi Muero! Mi Muero! She was very soon relieved by the use of ice and opiates.

5. A woman with the catamenia upon her eat heartily of ham and fowl, &c., one day, and early on the following morning vomited what she eat on the day before, still in an undigested state; and at the same time, she rejected by vomiting green and bitter matter, and voided stools acrid and hot, which were frequently repeated, and accompanied with violent cramps in the legs. Respiration was excessively oppressed; she had palpitation at the heart, and a painful sensation, attended with a feeling of impending death.

When this patient had a short respite of suffering, she complained that the relief was only momentary, and always followed by renewed and greater distress. Her tongue was somewhat furred; the lips preserved their natural redness, but the skin was cold, and at the wrist no pulse was felt. The flow of the catamenia was not interrupted during the attack. On the following day she was well.

6. In more intense instances than those above cited, we find the patient, whether male or fe-

* Rice is a standard article of diet in Lima. It is usually cooked with abundance of lard, and this probably is the reason why rice has the name of being very heavy, or difficult of digestion in hot weather.

male, with a cadaveric countenance, the sight obscured, the voice low and sunk, vomiting, evacuations, and cramps nearly incessant, a cold clammy sweat on the body, and the powers of life apparently about to become extinct.

The common curative plan of the vulgar in cholera, as it ordinarily appears among the natives, is short and simple. They at first freely give diluents, as warm water, or mallow water, with a little cream of tartar or tamarinds, until they consider that the patient has vomited and purged enough, or thrown off any undigested matter that may have been a source of irritation, and then they administer ice-water, which produces a powerfully sedative effect.

Opiates and ice-water conjoined are sometimes tried by several of the medical practitioners, as they always were by the writer, with unfailing success, in every case where the disease continued with urgency, after the bowels were sufficiently cleaned by the above simple means. But in cases like the first example above given, where the brain is greatly oppressed, an enema of warm water and sugar, hot fomentations succeeded by warm dry flannels to the feet, and embrocations to the belly, are useful, as, by producing a revulsion towards the lower extremities, and facilitating alvine evacuations, the head is relieved; and any warm diluent, as linseed water, rendered palatable with syrup of gum-arabic, is to be used instead of iced-drinks, until the stomach be cleared of all crude food and offending matter.

The death-like coldness of the patient does not often induce the practitioner to administer cordials and stimulants, or deter him from giving ice, of which Lima never wants a sufficient supply, regularly conveyed from the nearest ridges of the Cordilleras. The consequence of the seasonable exhibition of iced-drinks, especially in the summer months, is well known to be, that the stage of external coldness is shortened by the early removal of the painful sense of internal heat, which iced drinks are found to relieve, while they thus render the whole attack shorter and milder. Under this very ancient* yet simple treatment, the fever consequent on exhaustion, or that of reaction, never appears with a dangerous aspect; and I suppose that a good reason for this may be, that no stimulants or stomachics stronger than chamomile tea, or infusion of orange rind, are commonly used in the state of collapse; for it is obvious, that, if more active remedies were used in the state of exhaustion, these might again show their accumulated effects too powerfully, when the nervous energy and sensibility of the patient become more equally diffused, and the warmth of the surface restored.

It may be remarked, that when the patient is found in a state of cadaveric-like coldness, and when all the powers of life appear to be deeply concentrated within, then it is that he anxiously calls for ice or ice-water, which, after the first passages have been cleared, as already noticed, the practitioner freely allows as an agent, which long, popular experience has amply accredited as the most powerful of sedatives, and that upon which the Limenians uniformly rely, in the cholera with which they are so well acquainted, as one of the scourges of their climate and geographic situation.

After a patient under severe cholera had freely vomited undigested food, and after his strength had failed so that he began to sink under the violence of the vomiting, evacuations and cramps, I have known the infusion of guaco, administered by an intelligent, though non-medical friend, stop the progress of the disorder, and bring about salutary reaction in course of a few hours.

Hitherto I have spoken of cholera as seen in Lima, from my own individual experience and personal knowledge; but, as the subject is one of no ordinary interest, I will translate the sentiments of two native physicians, as published by themselves; and I know, from personal acquaintance with all the parties, that these statements from two proto-medicos are no more than a written expression of the general practice, long since approved by common observation among the vulgar, and regularly pursued, without any difference of opinion, by the different members of the profession, who at this day enjoy public confidence in the capital of Peru.

"Cholera, which is vulgularly called lipyria, is a common malady in our climate, in the summer season, by reason of the abuse of fermented drinks, fruits, and food, at a time when the digestive powers of the stomach are weakened by perspiration. These powers being diminished from the moment that muscular action ceases, through sleep and the repose of bed, it is at night that the disease makes its invasion.

"It begins with giddiness, followed by vomiting, and copious evacuations, of various appearance, cold sweat, cramps, and death, if not checked in its progress. Those who would like to guard against this dismal illness should avoid the excesses alluded to, and retire to rest with a light stomach; but should they find themselves burdened with aliment which they are unable to digest, or incommoded with symptoms of acidity, they should use means to reject the undigested aliment, exciting vomiting by aid of warm water, and irritating the fauces with the fingers, or with a feather; or,

* "In cholera, eorum quæ ejiciuntur suppressio mala est. Cruda enim sunt, quare nos oportet ea facile sponteque exeuntia libenter permittere; si non exeant incitare, aquam tepidam sorbitione dantes."

"Sin autem omnia antiqua stercora dejecta fuerint, et biliosi humores transierint, biliosusque vomitus, et distentio adsint, fastidium, anxietas, virium labefactio; tunc frigidæ aquæ cyathi duo, aut tres propinandi sunt ad ventris adstrictionem; ut retrogradus humorum cursus cohibeatur, utque stomachus ardens refrigeretur." Aret. Cappad. de Curat. Morb. Acut., lib 2, cap. 4.

in place of exciting vomiting, they may cause a pair of purgative clysters to be administered, and presently take a couple of cupfuls of warm water, with sugar and some stomachic, as chamomile flowers, theriaca, orange peel, &c.

"Should this practice have been omitted, and should cholera supervene remissly, or not in a sudden or violent degree, the indication is to dissolve and expel the humours of the stomach, taking abundantly, in form of drink, and by clysters, chicken water or other equivalent; and after the stomach is considered to be unloaded, then will be used the stomachic drink which we have pointed out.

"But if cholera has supervened with violence, and the patient has voided a great quantity of humours, upwards and downwards, the ready remedy by which to relieve him from his struggle is to make him drink iced-water, (no matter whether it be natural water or chicken-water,) alone, or in form of lemonade; and he may also swallow bruised or pounded ice." Unanue sobre el clima de Lima, p. 278-9.

Dr. Valdes, at p. 13–14 of his pamphlet, entitled Memoria sobre las Enfermedades Epedemicas, que se padecieron en Lima El ano de 1821, Estando Sitiada por el Eejereito Libertador, writes as follows:

"And although the disease, cholera, is almost endemic among us, in the said year many more sick were affected by it than in preceding years.

"It attacked some who had eaten too much, or drank spiritous liquors; and others without manifest cause. The dejections were at first bilious, and afterwards whitish or serous. These were promptly followed by intense thirst, burning heat in the interior of the abdomen, coldness in the extremities, pulse small and frequent, hiccup, cramps, and other ominous signs; but all yielded to ice, taken without delay.

"It is amazing to see the quickness with which the vomiting becomes suspended, the evacuations diminished, hiccup removed, and the pulse raised and regulated; and without any assistance except this, (viz. ice,) and that of our subacid paps or 'mazamorras,' as the only nutriment, those recover their vitality and strength, who but a few hours before presented a living and horrid image of death."

*Lipyria.*—The disorder designated by the Limenians under this name is not *cholera*, though Unanue, as we have seen, makes use of the term *lipyria*, as synonymous with *cholera;* and in some parts of the interior it is still used in this general sense, as we may have an opportunity to notice hereafter. At present, however, I will give an instance of what I have been taught to consider as the true lipyria in Lima.

A Zamba girl eat rice and melon with great relish, but the result very soon after was an attack of perfect lipyria, characterized by cold sweat, eyes red, and so full, that they appeared ready to start from their sockets, headach violent, and pain of belly insufferable.

As this form of disease is sudden in attack, and follows soon after the exciting cause has been applied, the usual treatment is to give warm water, and procure vomiting, by which the offending matter is rejected, and the paroxysm ended; but should the disorder go on to assume the character of a regular cholera, then the treatment is the same as in that disease.

(*To be continued.*)

---

THOUGHTS ON MEDICINE.

There are nearly ten thousand organs in the human body, and each of them has a multitude of parts, which are themselves divisible, until we arrive at atoms subject to molecular affinities. Then setting out from this point, ascending from harmonies to harmonies, from one organic sphere to another, we arrive at the *ensemble*, the whole, the sensitive and moral unity, the *I*. This is man. But of what does this vital and plastic force consist? What is the hidden bond, the primordial element, which generates this surprising variety of actions? The unknown quantity in this problem has not been discovered. All the parts of the body have life, but not *a life*, and yet they converge towards this unity with an admirable concord; every faculty terminates and loses itself in the abstract and hyper-organic faculty of personality. By what means does nature effect this great phenomenon? The triple scale of ignorance still covers our feeble eyes. What problems to resolve! What veils to lift! What depths to sound! You may now understand the splendour of Stenon's words: *Pulchra sunt quæ videntur, pulchriora quæ sciuntur, sed longe pulcherrina quæ ignorantur.*

—

You are irritated against a criticism; it annoys, it wounds you. Weigh it well, however: if it is by a fool, forget it; if it is by an envious man, forgive it; if it is by a severe friend, make use of it. In any case, remember that it requires as much talent to profit by a good crititique as to be able to do without it.

—

The town of Nancy gave *fêtes* to celebrate the recovery of its prince. "The Lorrainers struck two hundred gold medals with the arms of Nancy on one side, and those of M. de la Peyronnie on the other. He persisted in refusing to accept them; but not to disoblige such loyal subjects, he accepted an equal purse of silver medals." (*Eloge de la Peyronnie*, by Morand.) Suppose the same thing to occur at present, friendly reader, you have no doubt what would follow.*

* The author means, of course, that in the present day every one would take the gold. But do not immense competition and small gains almost force such conduct on the profession? To speak in our author's style, the hungry wayfarer snatches at a crab-apple, while the tenant of the gardens of Alcinous turns away, satiated, from peaches.—*Translator's note.*

